# The Search for an Efficient Black Fly Trap for Xenomonitoring of Onchocerciasis

**DOI:** 10.1155/2018/5902367

**Published:** 2018-07-03

**Authors:** Kenneth B. Otabil, Samuel F. Gyasi, Esi Awuah, Daniels Obeng-Ofori, Mario A. Rodríguez-Pérez, Charles R. Katholi, Thomas R. Unnasch

**Affiliations:** ^1^Department of Basic and Applied Biology, School of Sciences, University of Energy and Natural Resources, Sunyani, BA, Ghana; ^2^Department of Civil Engineering, Kwame Nkrumah University of Science and Technology, Kumasi, AR, Ghana; ^3^Office of the Vice Chancellor, Catholic University College of Ghana, Sunyani, BA, Ghana; ^4^Instituto Politécnico Nacional, Centro de Biotecnología Genómica, Reynosa, TAMPS, Mexico; ^5^Department of Biostatistics, School of Public Health, University of Public Health, University of Alabama at Birmingham, Birmingham, AL, USA; ^6^Global Health Infectious Disease Research, College of Public Health, University of South Florida, Tampa, FL, USA

## Abstract

Onchocerciasis is a neglected tropical disease that has plagued mankind for decades with pathologies that involve the eyes and the skin. The WHO and the global health community have earmarked the disease for global elimination by 2045. However, as control programmes shift focus from reduction of the burden of the disease to elimination, new tools and strategies may be needed to meet targets. Monitoring* Onchocerca volvulus *larvae in the black fly vectors is an important tool needed to monitor disease dynamics and certify elimination. For decades, human landing collections have been the sole means of acquiring vectors for monitoring of the disease. This procedure has been plagued with ethical concerns and sometimes the inability to harvest enough black flies needed to carry out effective monitoring. Since the 1960s, the WHO recognized the need to replace human landing collections but relatively few field studies have designed and tested alternative traps. This review article systematically discusses some of the key traps tested, their successes, and their challenges. It is the aim of the review to direct research and development focus to the most successful and promising vector traps which could potentially replace the human landing collections.

## 1. Introduction

Onchocerciasis (river blindness) is a neglected tropical disease (NTD) caused by the parasite* Onchocerca volvulus *and transmitted by riverine vectors of* Simulium* sp. or black flies. Currently the disease is still a major public health concern after years of both vector control and Mass Drug Administration (MDA) [1, 2]. It is a chronic cutaneous and ocular disease characterized by skin nodules, severe itching, and ocular lesions that can progress to partial or total blindness [3, 4]. It causes a great morbidity and is the second leading cause of blindness due to infectious disease worldwide. Globally, an estimated 37 million people are infected with* O. volvulus* and 120 million people are at risk [3, 4]. However, these figures are generally accepted to underestimate the true situation [5].

The WHO has earmarked the disease for global elimination by the year 2045 [6]. However, experts opine that new tools and strategies are needed for the elimination efforts to be successful. Monitoring of the prevalence of the infective larvae and biting rates as well as other entomological indices of the disease in the black fly vectors (a process called xenomonitoring or xenodiagnosis) has been and continues to be an important component of control programmes. However, more can be done to position xenomonitoring as a crucial tool in control programmes especially as global focus has shifted from reducing the public health burden of the disease to elimination. Some identifiable challenges associated with xenomonitoring of onchocerciasis include ethical concerns on the use of human landing collections (HLCs), the urgent need for vector traps to replace HLCs, and algorithms that link transmission dynamics of HLCs with trap collections (when replacement traps are developed).

In the roadmap of the WHO for the elimination of onchocerciasis, the prevalence of the infective stage of* O. volvulus* larvae in the black fly vectors is used as a major index for determining whether or not transmission has been successfully interrupted in an endemic community [7, 8]. The threshold for declaring that elimination has been achieved in a previously endemic area is when one can say with 95% confidence that there is less than one (<1)* O. volvulus *larva per 2,000 female black flies per endemic community. To satisfy this criterion, it is important to examine at least 6,000 flies by PCR (Pool Screen technique) in each endemic community [7–9]. Till date, getting such huge numbers of black flies has been difficult especially in hypoendemic and mesoendemic areas where densities of biting black flies are low or highly seasonal.

Currently, in global onchocerciasis programmes, the gold standard for vector capture is human landing collections (HLCs). However, this method has often fallen short of being able to trap the large numbers of black flies sufficient to demonstrate that transmission has been interrupted especially in communities where biting rates are low or highly seasonal. Moreover, there have been concerns about how ethical it is to station human beings in one particular place (sometimes with very high vector densities) for several hours and deliberately expose them to harm, in order to catch the black fly vectors. Of course, these volunteers receive prophylaxis and are also properly trained so they can catch the vectors, ideally, before the vectors get the chance to take a blood meal. However, experience from the field has shown that human volunteers often receive unexpected bites, in spite of all the precautions taken. Thus, the global community advocates for a replacement for HLCs and rightly so. There is therefore an urgent need for research and development focus on traps to replace HLCs, as the global focus in onchocerciasis continues to shift from control to elimination and, in some cases, postelimination surveillance.

The main aim of this review article is to discuss the various black fly vector traps that have been developed and tested on the field and their successes and failures and to identify gaps in their design. This will help direct research focus to the most promising traps that can be optimized as potential replacement for HLCs.

## 2. Methodology

### 2.1. Eligibility Criteria

Selection of the studies to include in this study was based on the following: (1) the study must be on xenomonitoring of river blindness; (2) the study must be published in English language; (3) studies must be on black fly vector traps; (4) studies must be published between 1970 and 2018. The exclusion criteria for the purpose of this review were (1) studies not connected to river blindness and (2) review articles.

### 2.2. Search Strategy

**Figure 1:**
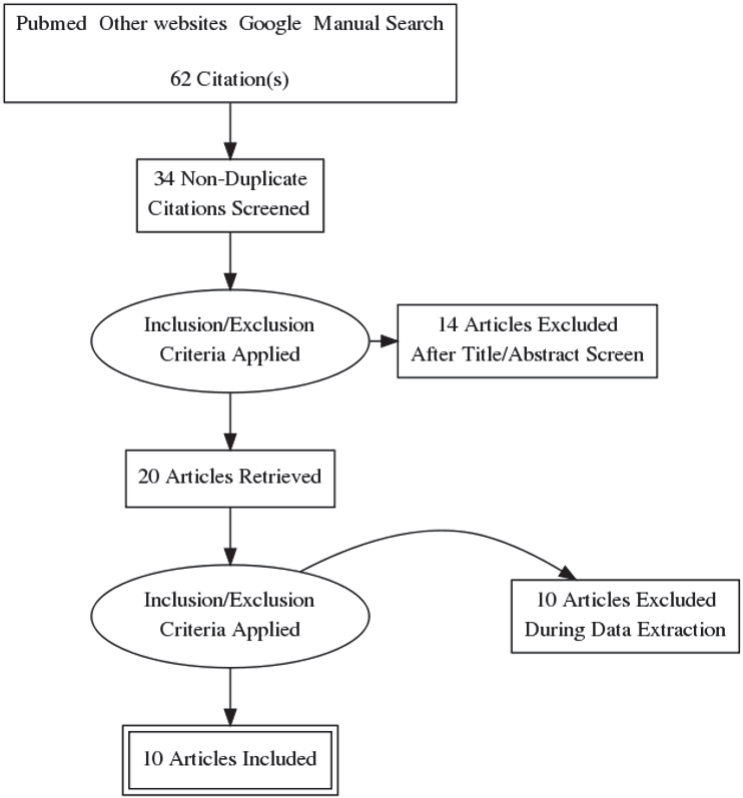
PRISMA diagram of publication flow.

The search was electronic, targeting studies that designed and tested traps to capture black flies. This produced 64 references but only 9 were included in the review using the inclusion/exclusion criteria (***see Figure 1***). Articles were extracted by electronic search from PubMed, NCBI, and other relevant journals. Various key words utilized in the search strategy included the following: onchocerciasis, river blindness, human landing catches, sticky traps, light trap, human silhouettes, Esperanza window traps, insect adhesives, and black fly. Various combinations of key words were made using “AND” and “OR” as Boolean operators. One (1) unpublished work which contained data which filled a very essential gap identified by this review work was also included.

## 3. Results

### 3.1. Results of the Performance of Light Traps as HLC Replacement

The first study reviewed is a study by Walsh (1978) in which light traps were tested during the wet and dry seasons [10]. They observed that flies in different physiological states behaved differently as gravid females and newly emerged flies appeared to be strongly phototactic. Based on this premise, light traps were developed with the goal of attracting the black flies towards the light and using the traps to capture them for analysis. The number of host seeking females attracted to the traps was however very few.

In a later study by Service (1979), light traps were tested for their ability to collect ovipositing* Simulium squamosum* populations in Ghana [11]. Interestingly, in their study, the investigators were able to collect as many as 14, 644 females and 2 males of* Simulium squamosum* in just 4 nights using Monks Wood light traps. The highest catch of 6,520 females was obtained in a single night with an ultraviolet light tube that had a one-second flash rate.

### 3.2. Results of the Performance of Sticky Traps as HLC Replacement

The first study on sticky traps reviewed was by Thompson (1976) where four (4) different black fly vector traps (the sticky trap, slat trap, the enclosure trap, and the fan trap) were investigated [12]. The design of the trial traps was aimed at testing the relative importance of sight, exhaled breath, and smell and their ability to attract* Simulium damnosum* senso* lato *(Diptera: Simuliidae) to the traps. In their study they found out that carbon dioxide gas emitted from inside the traps attracted two-thirds as many flies as a man exhaling normally inside the trap. In the forest, a trap baited with CO2 gas (250 cc per min) caught more flies than a similar, unbaited trap. The investigators also reported that when olfactory substances from the human skin were removed by vigorous washing and application of petroleum jelly, or by wearing impermeable clothing, the number of flies attracted to the traps greatly reduced. However, these reductions were not observed in the Sudan-savanna areas. They further reported that, in the forest areas, clothing worn by men for several days and used as bait attracted about 10 times more flies when compared to unworn clothing. The addition of CO2 gas produced a 4-fold increase in the attractiveness of worn clothing and an 18-fold increase in that of unworn clothing. In the Sudan-savanna, the catches of men positioned in front of warm rock surfaces were lower than those of men stationed on cooler, sandy surfaces. This suggests that human body heat may be an additional attractant factor [12].

Walsh (1980), working in Northern Ghana, also experimented on the ability of sticky traps to capture* S. damnosum* [13]. Generally, the number of black flies captured by the sticky traps was always lower than those obtained by HLCs. Moreover, the flies captured by these traps tended to disappear from the traps which were not emptied in a day. Colour choice studies showed that hunting flies were attracted to dark blue substrates. In all situations the most shaded surfaces of the traps were preferred by alighting flies. The presence of* S. damnosum* in the canopies of trees was demonstrated for the first time by this study. Flies were caught at heights above 6 m at three sites, the maximum height being 9.2 m, the height of the topmost trap. Among flies caught in the canopy of trees were nulliparous, parous, and gravid females as well as males [13].

### 3.3. Results of the Performance of Human- and Cattle-Baited Tents and Other Traps as HLC Replacement

**Figure 2:**
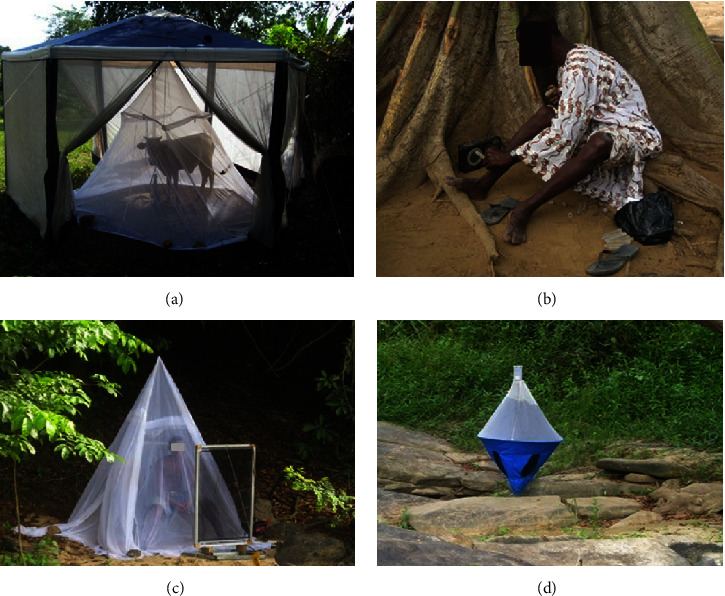
Methods used to collect host seeking female black flies. (a) Cow-baited tent. (b) Standard human landing catch. (c) Electric nets near a human protected by a mosquito net. (d) Biconical traps baited with human odour [Source: Lamberton* et al.*, 2014].

In 2014, Lamberton and his colleagues working in some communities in Ghana tested the efficiencies of human- and cattle-baited tents, human landing catches, electric nets, and biconical traps to capture black flies [14]. The traps used in collecting host seeking flies are shown in***Figure 2.***

The electric nets were selected based on preliminary tests that showed a lot of promise in their ability to capture tsetse flies and mosquitoes [15–18]. They had dimensions of 1 m × 0.5 m and were either alone or baited with a human volunteer or human odour-baited, black cotton sheet placed within the net [14]. However, the results showed that they were inefficient in trapping significant numbers of* S. damnosum senso stricto *as only 1 black fly (which was a male of the Beffa form of* S. soubrense, *a non-host seeking fly) was caught [14].

The human-baited tents in the study by Lamberton et al. (2014) were able to collect 22% of the total 9,916 host seeking female black flies compared to the 16% collected by cow-baited tents and 62% collected via HLCs [14].

**Figure 3:**
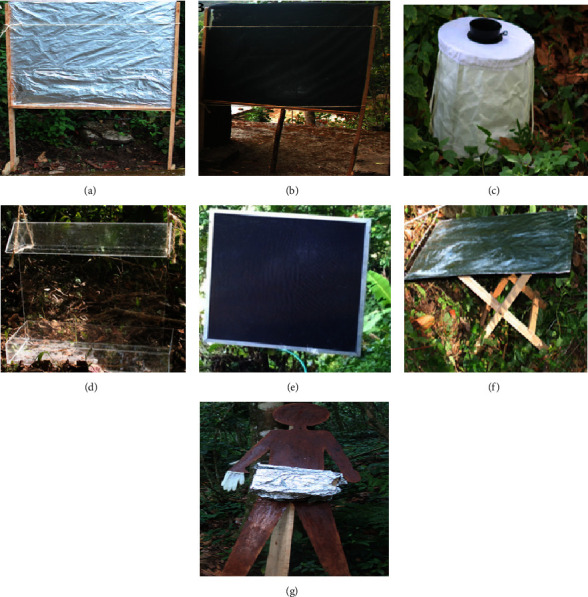
Traps evaluated by Rodriguez-Perez et al. (2013) for their potential in collecting* S. ochraceum s.l.* females in Mexico. (a) Vertical Bellac Plaque Trap. (b) Esperanza Window Trap (original). (c) BG Sentinel. (d) Clear Window Trap. (e) Esperanza Window Trap (acrylic/hanging version). (f) Horizontal Bellac plaque. (g) Human silhouette trap [source: Rodriguez-Perez* et al.*, 2013].

As part of efforts to develop a trap to replace human landing collections for the monitoring and surveillance of onchocerciasis transmission, Rodriguez-Perez et al. (2013) designed and tested different black fly vector traps for their ability to capture black flies. The tested traps were the Vertical Bellac Plaque Trap, the original Esperanza Window Trap, the BG Sentinel, the Clear Window Trap, a modified version of the Esperanza Window Trap (acrylic/hanging version), the Horizontal Bellac Plaque, and the Human Silhouette Trap** (*see Figure 3***). All the traps mostly targeted host seeking black flies [19].

Among the traps tested for their potential as HLC replacements, the Esperanza Window Traps (EWTs) were more promising than the other designs [19]. This trap consists of a square of black satin cloth sandwiched between two sheets of clear acrylic plastic. The surfaces of the trap were coated with Tangle Trap™ insect adhesive® to collect insects landing on the surfaces. The trap was baited with a combination of carbon dioxide (CO_2_) and a commercially available human attractant originally designed to attract* Aedes *mosquitoes, the BG lure® [19]. When tested on the field, a pair of EWTs collected numbers of* S. ochraceum s.l*. females similar to those collected by a team of vector collectors [19].

**Figure 4:**
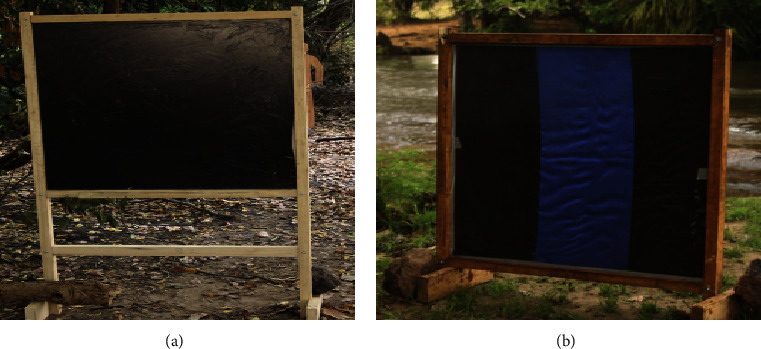
Esperanza trap designs: (a) Original design. (b) Optimized (short stripped) design [source: Toé* et al.*, 2014].

Toé et al. (2014) following up from the findings of Rodriguez-Perez et al. (2013) worked on optimizing the EWTs to enhance their ability to capture black flies and to determine if the results reported by Rodriguez-Perez et al. (2013) could be replicated in Burkina Faso [20].  Figure 4 shows the designs of the EWTs tested.

In their study, Toé and his colleagues (2014) optimized the EWT by changing the design and colour of the fabric used, the CO_2_ source, and the odour [20]. Again, the EWTs proved to be effective as they collected sufficient numbers of host seeking black flies similar to numbers collected by HLCs.

**Figure 5:**
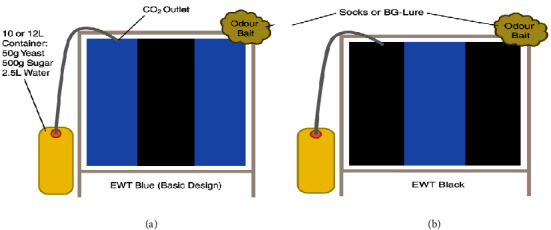
Blue and black trap designs showing position of CO_2_ and odour baits. Blue screens with a black vertical stripe (basic design) were used for all trapping experiments in Uganda. Black screens with a blue vertical stripes were additionally used in Tanzania (source: Hendy* et al.*, 2017).

In another study by Hendy* et al.* (2017), some further field testing of the EWTs was carried out [21] with the aim of optimizing the design for enhanced efficiency. The investigators built these traps based on previously published methods [19] to evaluate their efficacy and ease of use. They made modifications to the colour of the fabric used, the CO_2_ outlet, the odour bait, and the trap frame. They then tested to see how important each factor is in attracting black flies. The design of the traps tested is shown below (**Figure 5**). Blue fabric with a strip of black in the middle proved to be most efficient in collecting host seeking black flies.

Meanwhile, the prospects of the black fly traps included in this review are summarised in***Table 1***. Overall, though several traps have been tested, few have shown promise as replacements for HLCs.

## 4. Discussion

There have been several attempts to develop new vector traps, repurpose, or modify existing traps for the collection of host seeking, anthropophilic black flies. However, these efforts have been met with limited and sometimes conflicting success [19]. Some of the traps that have been used successfully to collect black flies in various physiological states included modified Challier-Lavessiere tsetse traps, sticky traps and silhouettes, BG-Sentinel traps, and light traps as well as other novel traps.

The study by Walsh (1978) showed little evidence to prove that light traps could be useful for the capture of blood engorged flies and for the routine monitoring of* S. damnosum* populations [10]. This is because the numbers of host seeking females attracted to the traps were low. The investigators thus advocated for further work on the use of these traps. Subsequently, the study by Service (1979) on light traps tested showed some promise as their light traps caught female* Simulium squamosum* populations either before or soon after oviposition. Unfortunately, the same traps placed in other locations either caught none or caught only a few black flies [11]. The above studies provide inconsistent evidence on the potential of light traps for the capture of black flies for disease monitoring. Therefore, it is important to investigate the factors that account for the differential performance of light traps in various locations. It is possible that different species of black flies react differently to light. Thus, studies should concentrate on diversity of black flies in various endemic communities and their reaction to light.

Two studies that tested the efficiency of sticky traps to capture host seeking female black flies were included in this review [12, 13]. Thompson (1979) confirmed that* S. damnosum* females relied heavily on smell as an attractant whilst sight and exhaled breath are lesser attractants of these black flies. The study by Thompson (1979) further showed that smell may be the only obligatory attractant, and it could act as a standalone stimulus [12]. These findings showed that it is possible to develop an effective vector trap which incorporates only smell stimuli as bait. But, for some savanna* S. damnosum s.l. *(presumably* S. damnosum s.s*.), neither smell nor exhaled breath appeared to be important attractants, and some other factors such as sight might be the dominant attractant in the area. However, more research is advocated as diverse species of* Simulium* react differently to visual cues, odours, light, and other stimuli. Additionally, it is noteworthy that the traps tested in this study had cumbersome setups and might be difficult to deploy in large numbers in control programmes. Another challenge is the fact that some of the setups still relied on human baits, and this is undesirable in a replacement trap for HLCs [12]. In the study by Walsh (1980), it was concluded that the sticky traps tested offered little promise as a means of monitoring population fluctuations [13]. This is because the setup was unable to attract sufficient numbers of anthropophilic female black flies which are needed for disease surveillance. The study however provided some valuable information (on attraction of black flies to the colour “blue”) which has subsequently been useful in the design of other black fly traps which have been much more successful.

In the study by Lamberton et al. (2014), the electric nets were not efficient as potential replacement of HLCs (at least not in its current design). This is because a potential replacement must be able to attract sufficient numbers of host seeking, female black flies in order to be relevant in monitoring the disease. However, only one male fly was caught by this trap. In order for electric nets to be relevant as HLC replacement, changes must be made to its design including changes to the colours used and odour baits as well as the provision of a CO2 source as all of these have demonstrably been important in attracting host seeking female black flies. Though the human-baited traps showed some promise, it still lacks the capacity to fully resolve issues of ethical concerns raised with the use of humans as baits as encountered in the implementation of HLCs. Again, the setups of both the human- and cow-baited tents make them cumbersome to implement in the field on a large scale. There is therefore the need to look for more robust trapping methods which are efficient, eliminate the need for using humans as baits, and are also relatively easy to deploy on the field.

In the study by Rodriguez-Perez et al. (2013), EWTs were the most promising. The EWTs collected black fly numbers similar to that of HLCs [19]. Aside from this, a major advantage of the EWTs over HLCs is the fact that several of the EWTs can be deployed in endemic communities and managed effectively by one or two persons. This means that the collective catches of these traps can exceed that of HLCs. This advantage has been demonstrated by Rodriguez-Perez et al. (2014) in a community-based trapping programme to collect* Simulium ochraceum senso lato. *The results from their study demonstrated that EWTs may be effectively operated by one or two minimally trained residents of formerly endemic communities, resulting in the collection of sufficient numbers of flies to verify interruption in transmission of onchocerciasis. They concluded that the EWT trap represents a viable alternative to using HLCs for the verification of onchocerciasis elimination [22].

Additionally, since the EWTs do not employ humans as baits, the need to seek informed consent of volunteers and other ethical concerns raised against the HLCs is not encountered in the deployment of EWTs. Going forward, research in this regard should focus on investigating the efficiency of the EWTs in different places to determine if they are effective in trapping different black fly species. To this end, Toé et al. (2014) further sought to optimize the design of the EWTs by changing the colours of the fabric used and testing in other locations apart from the originating country. They demonstrated that black fabrics with blue strips were better at attracting black flies than fabrics which were entirely black.

In the study by Hendy et al. (2017), the EWTs were tested in other locations apart from the originating country (Mexico). This was necessary because various countries have diverse species of black flies which react differently to diverse stimuli. In order for a trap to replace HLCs, it is desirable that it is universal in application and deployment. The results from the studies by Hendy et al. (2017) showed that, in Uganda, the traps worked efficiently for the collection of* Simulium damnosum*, the black fly species primarily responsible for onchocerciasis transmission in Sub-Saharan Africa. However, the traps were less effective at collecting the same species in Tanzania. They opined that black fly behaviour and response to traps will probably vary from one country to another and that though the EWTs showed promise for black fly collections, further research and development were needed to determine how broadly they can be used [21].

Other investigators in a parallel research demonstrated the usefulness of EWTs as black fly control tools when deployed in large numbers in an endemic community [23]. This is an added advantage of EWTs and can potentially be exploited to reduce the numbers of black flies in endemic communities. However, there is the need for more research on the ability of EWTs as reported by the original developers. If proven to be effective in several other trials, the EWTs can offer an eco-friendly, more direct, less expensive, and more robust vector control tool which can be deployed whilst community directed treatment with ivermectin is ongoing.

However, some questions still beg for answers with regard to the usage of EWTs and there is need for further research in this area. The first challenge encountered is that some authors report difficulties in the availability of the insect adhesives [21] used to trap the black flies in EWTs. Also the challenge with the cost of these adhesives even when they are available and the difficulties with application have been discussed [24]. A solution to this challenge may be to develop local alternatives using locally available materials so that the problem with cost and availability of the commercially manufactured adhesives can be eliminated.

Another challenge with the use of EWTs is the need for optimization and standardisation of the baits used in the traps. To tackle this, Young and colleagues (2015) set out to identify human semiochemicals attractive to the major vectors of onchocerciasis. They analysed human sweat compounds using GC-MS and compounds common to three individuals were identified. The common compounds identified as well as others previously identified as attractive to other hematophagous arthropods were evaluated for their ability to stimulate and attract the major onchocerciasis vectors in Africa (*Simulium damnosum sensu lato*) and Mesoamerica (*Simulium ochraceum s.l.)*. They found out that both species of vectors are attracted to ammonium bicarbonate and acetophenone [25]. Further research must focus on finding out more semiochemicals attractive to black fly vectors, which could then be utilized to produce black fly specific lures. This will help to optimize the trap for capturing of host seeking, female black flies which are much needed for surveillance of the disease.

Moreover, there is the need to develop algorithms to relate EWTs collections to human biting rates and infective rates. This is important because all calculations and projections in onchocerciasis control simulation models including ONCHOSIM rely on entomological indices estimated from vector catches from HLCs. It is thus important to develop new algorithms to relate the transmission dynamics of vectors caught by EWTs to that of HLCs. The foundation to solving this problem was laid by Katholi (2013; unpublished data) who used Poisson regression analysis of trap data to develop a stochastic model that related EWT trap collections to HLCs in Mexico [26]. Notably, the predominant species of black fly vectors in Mexico (as is generally the case in Mesoamerica) is* Simulium ochraceum s.l. *This means that the model developed by Katholi (2013; unpublished data) could be applied in control programmes in Mesoamerica. Recently, another model was developed by Loum et al. (2017) in Uganda, where the major black fly vector is* Simulium damnosum s.l. *[27] as is generally the case in Africa. The models by Katholi (2013; unpublished data) and Loum et al. (2017) describe the relationship between EWTs collections and HLCs in both Africa and Mesoamerica. Further testing of these models is desirable in order to demonstrate their robustness and possible usage in control programmes.

It is worthy of mention that no studies published in the 1990s and early 2000s on black fly vector traps were retrieved during the literature search for this review. Around this period, the Onchocerciasis Control Programme relied solely on HLCs for trapping vectors for disease surveillance. Onchocerciasis was a huge public burden and research focus was on reducing the public health burden. Therefore, global research efforts were more focused on getting antifilarial drugs which could help to reduce the burden of the disease. However, with the “wonder drug” ivermectin proving to be highly effective in interrupting transmission and antibiotics like doxycycline complementing treatment of the disease, control focus has shifted from reducing the burden of the disease to eliminating it globally. There is therefore a renewed interest in finding a replacement for HLCs as many endemic communities approach elimination of the disease.

## 5. Conclusion

In the face of global elimination efforts and targets, there is an urgent need to develop vector traps which can effectively replace HLCs in disease monitoring and surveillance. Several traps have been tested for their potential to capture host seeking black fly species for disease monitoring [10–14, 19–21, 23, 24]; however the most promising of such traps in recent times is the EWTs. These traps have clearly demonstrated promise in their ability to replace HLCs and may provide an added advantage as a means to control black fly populations if they are deployed in large numbers in endemic communities.

However, some gaps in the design of the EWT must be addressed if it is to become an optimal replacement for HLCs. Research should be carried out in several other countries apart from the ones which have been investigated by earlier researchers (Mexico, Tanzania, Uganda, and Burkina Faso). This is because different species of black flies in different locales react differently to visual cues, odours, light cues, etc. In order for the EWT to successfully replace HLCs it must be effective in capturing different species of black flies and more research should focus on this area.

Research must also focus on optimizing the design of the EWTs. In this regard, it is important to test for the effect of colour, size, CO_2,_ odours, etc., on the efficiency of the trap. More so, challenges with the availability and cost of the insect adhesives must immediately be addressed by researching on getting local alternatives which may be easier to prepare.

## Figures and Tables

**Table 1:** Summary of tested black fly traps and their prospects as replacements for HLCs.

**Author/Date of Publication/Reference**	**Name of Black fly trap tested**	**Ability to attract anthropophilic females (YES/NO)**	**Prospect as Replacement of HLCs**
Walsh (1978) [10]	Light traps	NO	Need for more research in the area

Service (1979) [11]	Light traps	YES	Need for research in this area

Thompson (1976) [12]	Sticky traps, Fan traps, enclosure trap, slat trap	YES	Vital findings on the importance of smell as attractants

Walsh (1980) [13]	Sticky traps	YES	Offered little promise in terms of replacing HLCs

Lamberton et al (2014) [14]	Cow-baited tents, human odour baited tents, biconical traps, and electric traps	YES (human odour baited tents showed most promise)	Setup too cumbersome. However, it demonstrated importance of smell as attractants in traps

Rodriguez-Perez et al (2013) [19]	Verticle Bellac plaque, Esperanza Window Trap (original), BG Sentinel, Clear Window Trap, Esperanza Window Trap (modified), Horizontal Bellac Plaque and Human Silhouette Trap	YES ( the Esperanza Window Trap was the most promising)	EWTs though promising, still need some optimization

Toé et al (2014) [20]	Esperanza Window Traps (original and modified designs)	YES	EWTs though promising needs some optimization

Hendy et al (2017) [21]	Esperanza Window Trap (original and modified)	YES	EWTs though promising needs more optimization and testing in other countries
